# A Few-Shot Learning-Based EEG and Stage Transition Sequence Generator for Improving Sleep Staging Performance

**DOI:** 10.3390/biomedicines10123006

**Published:** 2022-11-22

**Authors:** Yuyang You, Xiaoyu Guo, Xuyang Zhong, Zhihong Yang

**Affiliations:** 1 Beijing Institute of Technology, School of Automation, Beijing 100081, China; 2 Department of Electrical and Computer Engineering, Technical University of Munich, 80333 Munich, Germany; 3 Institute of Medicinal Plant Development, Chinese Academy of Medical Sciences and Peking Union Medical College, Beijing 100193, China

**Keywords:** few-shot learning, generative adversarial network, single-channel electroencephalogram, sleep stage classification

## Abstract

In this study, generative adversarial networks named SleepGAN are proposed to expand the training set for automatic sleep stage classification tasks by generating both electroencephalogram (EEG) epochs and sequence relationships of sleep stages. In order to reach high accuracy, most existing classification methods require substantial amounts of training data, but obtaining such quantities of real EEG epochs is expensive and time-consuming. We introduce few-shot learning, which is a method of training a GAN using a very small set of training data. This paper presents progressive Wasserstein divergence generative adversarial networks (GANs) and a relational memory generator to generate EEG epochs and stage transition sequences, respectively. For the evaluation of our generated data, we use single-channel EEGs from the public dataset Sleep-EDF. The addition of our augmented data and sequence to the training set was shown to improve the performance of the classification model. The accuracy of the model increased by approximately 1% after incorporating generated EEG epochs. Adding both the augmented data and sequence to the training set resulted in a further increase of 3%, from the original accuracy of 79.40% to 83.06%. The result proves that SleepGAN is a set of GANs capable of generating realistic EEG epochs and transition sequences under the condition of insufficient training data and can be used to enlarge the training dataset and improve the performance of sleep stage classification models in clinical practice.

## 1. Introduction

**Figure 1:**
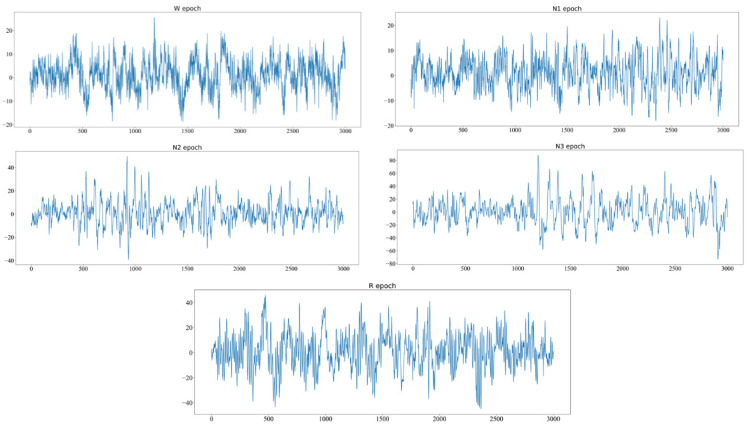
The EEG of different sleep stages according to the AASM manual. From top to bottom and left to right are W, N1, N2, N3, and REM.

As a restorative process, sleep plays a critical role in maintaining physical and mental health [1]. Monitoring the process of sleep is vital to people’s health and diagnosing sleep disorders. In the field of sleep science, experts measure the quality of sleep by observing electrical activity recorded by sensors attached to various parts of the body. These signals are reflected in a polysomnogram (PSG) [2]. Human sleep processes can be classified into different stages according to sleep manuals, such as those by Rechtschaffen and Kales (R and K) [3] and the American Academy of Sleep Medicine (AASM) [4]. For instance, the stages of sleep are divided into awake (W), rapid eye movement (REM), and non-rapid eye movement (NREM). Stage W refers to the state of wakefulness in early sleep. In the REM stage, the eyes move rapidly, and the level of brain activity is basically the same as in the awake stage. In the NREM period, the muscles of the whole body are relaxed, the heart rate and breath slow down, and no eye movement appears. NREM is divided into stages S1, S2, S3, and S4 in R and K and stages N1, N2, and N3 in the AASM manual. The EEGs of different sleep stages are shown in Figure 1.

Sleep staging manually based on sleep manuals is a time-consuming approach for sleep experts, so recent studies have focused on developing machine learning methods to automatically classify sleep stages. Some of these studies have aimed to utilize a number of algorithms to extract features from PSG signals, such as wavelet transform [5] and empirical mode decomposition [6]. Then, they use the extracted features as the input of the classifier to train models, such as random forest [7] and ensemble support vector machine [8]. Other studies have constructed deep neural networks (DNNs) to automatically extract features from raw data. The performance of DNNs has been proven to be effective in previous studies. Most of these studies [2,9,10] are based on convolutional neural networks (CNNs) and recurrent neural networks (RNNs). Refs. [11,12] introduced attention mechanisms into sleep staging. Ref. [13] proposed a graph-temporal fused CNN model. An increasing number of DNNs with different structures have been successfully used in sleep stage classification tasks, as shown by the studies mentioned above.

Most current deep learning methods rely on large-scale training data to reach a fine generalization performance. However, acquiring PSG data and labeling samples is expensive and time-consuming. Thus, models based on single-channel EEGs and few-shot training algorithms are becoming popular topics in the field of sleep staging [2,11,14].

Generative adversarial networks (GANs) have produced groundbreaking results for the generation of realistic images [15]. In recent years, GAN has also been used for EEG data augmentation. Most state-of-the-art (SOTA) EEG generation methods serve the field brain–machine interface (BMI) [16] and emotional recognition [17]; only a few studies have applied GAN to sleep staging tasks. Ref. [18] proposed five EEG data augmentation methods for solving the class imbalance problem in sleep staging; however, the lack of sleep data and data variety is still a problem. Compared with sleep staging tasks, the length of the EEG utilized in these tasks (around 2 s) is much shorter than the 30 s EEG epoch and overnight PSG in sleep staging. Furthermore, for brain–computer interfaces, there are as many different EEGs as there are control signals. However, EEGs for sleep staging have five classes according to the AASM manual. Therefore, it is hard to transfer EEG generation methods for BMI and emotional recognition to the generation of sleep EEG epochs.

This study introduces a data augmentation method based on GANs for sleep stage classification. The main contributions of our research are as follows:

(1) We propose a set of EEG-oriented progressive Wasserstein divergence GANs (WGAN-div) [19] that can adapt to sleep data and generate EEG epochs with few real data. The model can generate realistic 1D EEG epochs corresponding to different sleep stages and push the accuracy of the sleep staging model from 0.775 to 0.804.
(2) We generated stage transition sequences based on a relational memory (RM) generator [20], which was used to generate a long text. This scenario is similar to stage transition sequence generation, and thus, we propose a few-shot learning-based model to generate plausible sequences such that the generated samples can be used in the training of the models based on RNNs [21], which have been proven to be capable of extracting sequential features from EEG data, thereby further pushing the accuracy of classification model from 0.804 to 0.831.
(3) We evaluated our GANs by feeding both real data and EEG epochs and sleep stage transition sequences generated by us into a sleep staging model. In addition, we adopted the 1-NN method to ensure the efficiency of our GANs. The results showed that our GANs are capable of generating representative EEG epochs and plausible sleep stage transition sequences. With the help of the augmented data, the accuracy of the sleep staging model improved significantly after training with only a few samples.

## 2. Materials and Methods

### 2.1. Datasets

**Figure 2:**
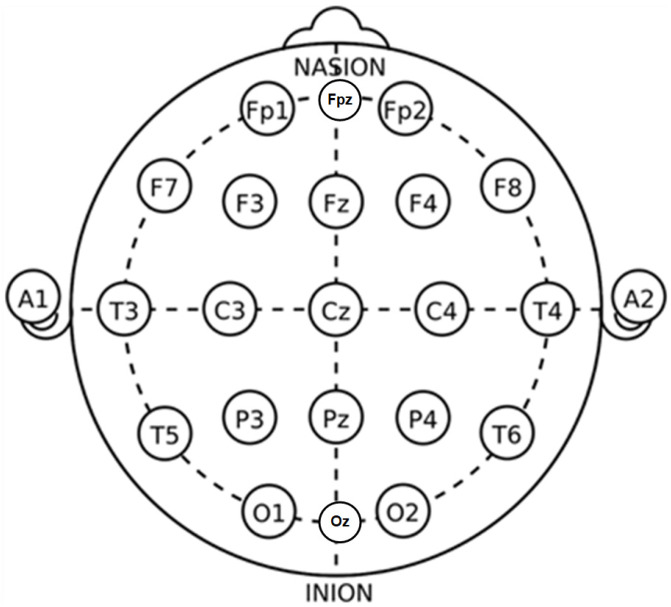
The position of electrodes of EEG acquisition system. The letters F, T, C, P, and O refer to the electrodes placed in the frontal, temporal, central, parietal, and occipital lobes, respectively.

We evaluated our method on the publicly available dataset SleepEDF [1]. There are two subsets in this dataset, Sleep Cassette (SC) and Sleep Telemetry (ST), which focus on the age effects and temazepam effects on sleep, respectively. Each recording in this dataset is composed of two EEG channels (Fpz-Cz and Pz-Oz), one electromyogram (EMG) channel, one electrooculogram (EOG) channel, and one oro-nasal respiration signal. We selected the Fpz-Cz channel EEG recordings from subset SC as our training and validation data. Here, Fpz-Cz and Pz-Oz refer to the positions of the electrodes. The placement of the electrodes is shown in Figure 2.

The recordings were divided into 30-s epochs, and each epoch was manually categorized into one of the eight classes in accordance with the R and K standard by sleep specialists. Most of the sleep staging models, including our baseline classification model, adopt a 5-class classification standard in the AASM manual. For convenience in comparing the classification performance, we selected AASM as the standard and maintained consistency with the references, merging S3 and S4 into N3. Moreover, we included W epochs of 30 min before and after the sleep periods. We also left out the MOVEMENT and UNKNOWN stages, as they had no relationship to our classification tasks [4].

### 2.2. EEG Epoch Generation

The GAN framework is composed of two networks; one is the discriminator and the other is the generator [15]. The discriminator is trained to judge whether the input data are real data or fake data. The generator tries to generate samples that tend to be recognized as real by the discriminator, and its input is a latent noise variable *z*. Hence, the generator is forced to produce better samples by the discriminator [16].

The main disadvantage of GAN is the instability problem of the discriminator. It may become possible for the discriminator to recognize only a limited range of input distribution modes as real. Therefore, the generator will only produce a limited range of outputs. This phenomenon is called modal collapse, which has been the topic of many studies [22,23,24,25].

Wasserstein GANs (WGANs) and their improved version [23] show promising advances in training stability and mode diversity. The Wasserstein distance introduced by WGAN is a metric that can measure the distance between any two distributions, that is, how similar any two distributions are. WGAN aims to minimize the Wasserstein distance between real data and fake data distributions; however, the original form of the Wasserstein distance is hard to compute, so the dual form of the Wasserstein distance is proposed, which requires a strict 1-Lipschitz constraint. WGAN-div introduced Wasserstein divergence, which does not require the 1-Lipschitz constraint, to solve this problem [19]. Thus, the losses of the discriminator and generator are:

(1) $$\begin{array}{c} L_{D}=E_{x_{f}\sim q\left(x\right)}\left[D\left(x_{f}\right)\right]-E_{x_{r}\sim \tilde{p}\left(x\right)}\left[D\left(x_{r}\right)\right]+kE_{\hat{x}\sim r\left(x\right)}\left[\|\nabla_{\hat{x}}D{\left(\hat{x}\right)\|}_{2}^{p}\right] \end{array}$$

(2) $$\begin{array}{c} L_{G}=E_{x_{r}\sim \tilde{p}\left(x\right)}\left[D\left(x_{r}\right)\right]-E_{x_{f}\sim q\left(x\right)}\left[D\left(G\left(z\right)\right)\right] \end{array})$$

where $\tilde{p}\left(x\right)$ is the real data distribution, $q\left(x\right)$ is the fake data distribution, and $r\left(x\right)$ is a distribution with the same sample space as $\tilde{p}\left(x\right)$ and $q\left(x\right)$. $x_{f}\sim q\left(x\right)$ refers to the generated data $x_{f}$ following the distribution $q\left(x\right)$. $x_{r}\sim \tilde{p}\left(x\right)$ refers to the real data $x_{r}$ following the distribution $\tilde{p}\left(x\right)$. $\hat{x}$ are the random interpolates between the real and fake samples.$\;D\left(x\right)$ means the output of the discriminator, while x is the input of the discriminator. Similarly, in Equation (2), $G\left(z\right)$ is the output of the generator, while z is the input of the generator. Therefore, $D\left(G\left(z\right)\right)$ means that the output of the generator $G\left(z\right)$ is the input of the discriminator. p and k  are hyperparameters, which are determined by experiments. In our model, p and k  were set to 2 and 6, respectively.

The architectures of the generator and discriminator and the training algorithm were based on those of ConSinGAN [26]. ConSinGAN proposed several methods to improve the generation performance and accelerate the training, such as multistage training, learning rate scaling, and an improved rescaling pyramid [27].

**Figure 3:**
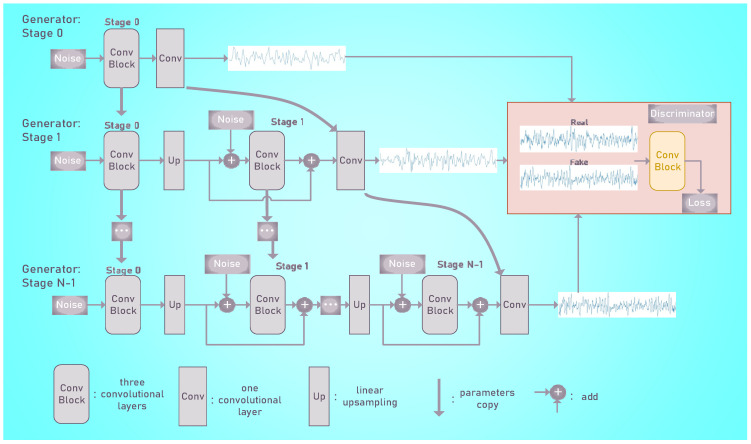
An overview of a model of EEG epochs generation. The training starts at stage 0 with a small generator and small sample resolution. With increasing stages, both the generator capacity and the sample resolution increase. Gaussian noise is used as additional noise.

Multistage training started on a low resolution in the first few iterations, learning a mapping from noise to a low-resolution EEG epoch (see “Generator: Stage 0” Figure 3). The generator size was increased by adding three additional convolutional layers once the training of stage n has converged. There was a residual connection between the original upsampled features and the output of additional convolutional layers [28]. This process was repeated N times until the desired output resolution was reached. Note that additional noise was added to the features at each stage [29,30] to improve diversity.

In the default setting, we trained the last three stages of a generator to avoid overfitting. The parameters of the discriminator are initialized to the parameters of the former stage instead of reinitializing to random values, which can accelerate the training process.

Assume the resolution of stage *n* is $x_{n}$, $x_{N}$ is the output size of the last stage. Then $x_{n}$ is defined as $x_{n}=x_{N}\times r^{\left(\frac{N-1}{\log\left(N\right)}\right)*\log\left(N-n\right)+1}$, *n* = 0, 1, …, *N* − 1. There are 4 stages in our training process (stages 0, 1, 2, and 3), so *N* is the number of growing stages set to 4. r is a rescaling factor defined as:

(3) $$\begin{array}{c} r=\sqrt[{N-1}]{\frac{S_{n}}{S_{r}}} \end{array}$$

In Equation (3), $S_{r}$ is the size of the real samples, which equals 3000, and $S_{n}$ is the size of the latent noise vector, which is 100. Therefore, the output sizes of our 4 stages were 100 (stage 0), 230 (stage 1), 965 (stage 2), and 3000 (stage 3).

Note that the training samples were mean-normalized EEG epochs since the normalization was capable of accelerating the convergence of the model [31], so the amplitudes of generated samples were also between −1 and 1. To generate samples with realistic amplitudes, we used two strategies for denormalization. The first strategy was recording the means, maximums, and minimums of training samples as sets of normalization factors and then randomly selecting a set of normalization factors to denormalize the generated sample. The second strategy was generating new sets of normalization factors through SMOTE [32] on the basis of the sets of normalization factors of real samples. Then, we denormalized the generated samples with generated sets of normalization factors.

### 2.3. Stage Transition Sequence Generation

The transition of sleep stages is periodic, with each sleep cycle taking about 90 min [33]. According to the rules of sleep stage transition, sleep experts can use information from the past and future to classify the current sleep stage. Many automatic sleep stage classification methods have also attempted to use RNNs, which are capable of considering information from the past, to improve classification performance. In reality, epochs are manually sliced from a long EEG signal segment and are correlated to the epochs before and after them. However, EEG epoch generation can only generate independent epochs. Hence, the generated samples cannot be applied to the training of the models based on RNNs.

To make the generated samples available in the training of models based on RNNs, we proposed stage transition sequence generation. The stage transition sequence generator we utilized was the generator of RelGAN by Weili, N. et al. [20] based on relational memory (RM), which was used to generate a long text. In our scenario, the sequences only consisted of five different sleep stages, so we only needed a minor modification of the vocabulary to make the model available for the task.

**Figure 4:**
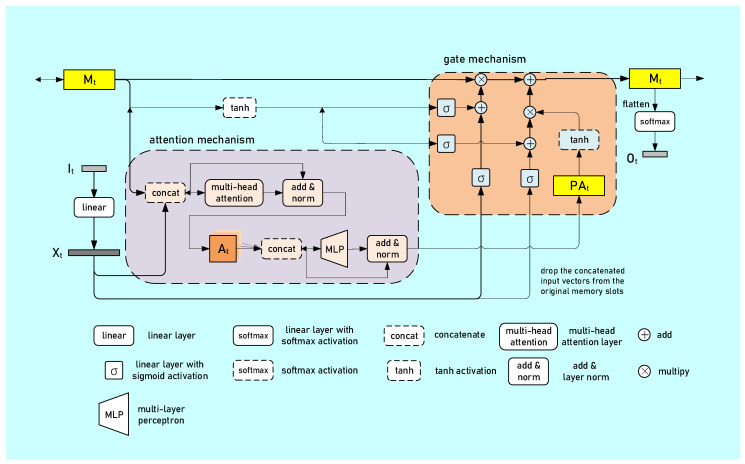
Overview of the relational memory generator. M_t_ is the memory matrix, the modules in the purple block constitute the attention mechanism, and the modules in the orange block constitute the gate mechanism.

The architecture of the generator is shown in Figure 4. The RM generator mainly consisted of attention and gate mechanisms.

An attention mechanism is capable of extracting self-attention weights from the input so that the model can pay more attention to the important part of a sequence. In this mechanism, we used a multi-head attention layer [34] with skip connection and layer normalization [35] to obtain the attention-weighted memory A. We further extracted the post-attention weighted memory PA from A using an MLP with skip connection and layer normalization.

A gate mechanism was specifically introduced to tackle the long dependence problem [36] in the RNN. Note that we only used the forget gate and input gate in this work. The forget gate (the left branch) was used to electively attenuate the useless information from the past, and the input gate (the right branch) was used to select important information from the current time step to update the memory.

Stage transition sequence generation is a much simpler task than long text generation, and through experiments, we found that adversarial training in RelGAN was trivial to the performance improvement in this scenario. On the basis of our experiments, the training of the RM generator was only supervised.

We trained the model with maximum likelihood estimation (MLE) loss, which is defined as Equation (4) below.

(4) $$\begin{array}{c} L=\frac{1}{N}\overset{T}{\underset{t=0}{\sum }}log\left(-O_{t,I_{t+1}}\right) \end{array}$$

In Equation (4), N denotes the length of the input data. *I_t_* denotes the true stage at the *t*-th time-step, and the RM generator is trained to predict the next possible stage (i.e., $O_{t}$) on the basis of $I_{t}$. However, in sequence generation, the input sequence is not given, except for *I*_0_, which denotes the start letter. Hence, the output at the *t*-th time-step is supposed to be the input of the next time-step (i.e.,$\;I_{t+1}$).

A generated stage transition sequence can be seen as a target sequence in training. For each entry of the generated sequence (i.e., a single sleep stage), we randomly picked an EEG epoch generated by the corresponding EEG epoch generator to synthesize a sequence of EEG epochs. The EEG epoch sequence can then be used to train the classifier as an augmented sample.

## 3. Results

### 3.1. Choice of Hyperparameters and Metrics

For EEG epoch generation, the detailed hyperparameters of the generator, discriminator, and training algorithm are shown in Table 1. In the hyperparameters of the generator and discriminator (Table 1), Conv 9 denotes a convolutional layer whose kernel size was 9 and whose stride was 1. Upsampling denotes the linear upsampling method. Norm./Act. Denotes the normalization and activation layers following the corresponding convolutional layer, respectively. LreLU (0.05) denotes the leaky ReLU activation, whose alpha was 0.05. The output size 32 × 100 denotes the output with 32 channels, and the size of the vector of each channel was 100; 32 was also the number of output channels of the corresponding convolutional layer.

Table 2 summarizes the hyperparameters used in the stage transition sequence generation. The choice of the sequence length (i.e., 180) was based on the duration of a human’s single sleep cycle, approximately 90 min. Limited by the model’s capability, the generation of the stage transition sequence for a whole night was difficult to achieve, and the quality of generated sequences was unsatisfactory. Compared with other shorter sequence lengths, the generated 180-long sequence also had better performance in tests.

We evaluated the performance of SleepGAN by evaluating the model DeepSleepNet trained with our generated data using the overall accuracy (ACC), macro-averaging F1-score (MF1), per-class F1-score (F1), and Cohen’s Kappa coefficient (k). The ACC and MF1 were computed as follows:

(5) $$\begin{array}{c} ACC=\frac{\sum_{c=1}^{C}TPc}{N} \end{array}$$

(6) $$\begin{array}{c} MF1=\frac{\sum_{c=1}^{C}F1c}{C} \end{array}$$

where *C* is the number of sleep stages, which is 5, and *TP_c_* is the true positive of class *c*, which indicates the number of correctly recognized class examples. *N* is the total number of test samples. *F*1*c* is the per-class F1-score of class *c*, which is calculated by treating a single class as a positive class and merging all other classes into a negative class.

### 3.2. Data Augmentation

We tested the data and sequence generated by our GANs on the model DeepSleepNet 2. The details of our eight tests are as follows:

Test 1-1 was constructed from real data collected from one patient, which was the benchmark of our experiment. Test 1-2 trained with real data from one patient along with augmented data generated according to this patient’s data in one night. Test 2-1 used real data of 1 patient and the other two patients’ two-night data for training. Test 2-2 used the same real data as test 2-1 and augmented data generated from the patient’s one-night data.

Test 2-1-1 trained with both augmented data and data and sequences from one patient’s one-night data and sequence. Test 2-1-2 used augmented data and sequences generated from the data and sequences of one patient for one night, as well as real data from this patient. Test 2-2-1 was performed with real data and sequences of one patient in one night and two other patients in two nights and augmented data from this patient. Test 2-2-2 used real data from one patient in one night and two patients in two nights, adding both augmented data and the augmented sequence derived from the data and sequence of this patient for training.

Table 3 shows the classification results of using real data and augmented data as a training set. When we chose to train our model with more real data, we found that the performance was better than that of the original test 1-1. On the basis of test 2-1, we trained our model with augmented data generated by our EEG generator, and the model achieved an accuracy, macro F1, and k of 0.804, 0.717, and 0.716, respectively, thus performing the best in our four tests.

We concluded, on the basis of the comparison of the results of our four different tests, that the larger the dataset used for training, the more powerful the model will be. When the amount of real data in different training sets was equal, the model trained with data generated by our EEG-epoch generator was more effective.

### 3.3. Sequence Augmentation

Based on the data augmentation experiment mentioned above, we added augmented sequences to our training dataset. Table 4 shows the scores of our network and the details of our test.

As shown in Table 4, the overall metrics were much higher than when only data augmentation was used to train the model. Therefore, the introduction of sequences containing past and future information can improve the classification scores of the model. Further improvement of the model’s performance can be achieved through the use of sequences generated by our SleepGAN. Augmented sequences can enhance the diversity of sequences as well as the generalization and robustness of the model.

### 3.4. Data Distribution Evaluation Via 1-NN Classifier

The K-nearest neighbor (k-NN) classification algorithm [38] is one of the classical methods in classification tasks. K-nearest neighbor means that each sample can be classified into the major class of k values that are closest to it. The 1-NN (1-Nearest Neighbor) classifier is a specific form of the k-NN classifier. If we set k = 1, then the k-NN classifier is a 1-NN classifier. The 1-NN-based two-sample test is a sample-based evaluation metric for GANs. In two-sample testing, the 1-NN classifier is utilized to determine whether two distributions are similar to each other [39,40].

We introduced the 1-NN classifier to test the similarity between the distribution of the data generated by our GANs and that of real data. It works by calculating the Euclidean distance of two EEG epochs and classifying both generated data and real data to the category of the nearest sample. We computed the accuracy of a 1-NN classifier trained on real EEG epochs and generated EEG epochs with positive labels for real data and negative labels for generated data. In this scenario, the 1-NN classifier should obtain an accuracy of around 50%. The 50% accuracy of the 1-NN classifier means that the generated data are very similar to real EEG signals of the same sleep stage and can hardly be distinguished from the real one, which indicates that the two distributions match and the GANs perform well. The average and variance accuracy of our 1-NN classifier is shown in Table 5.

In Table 5, the accuracy of the 1-NN classifier is presented. To better show the distribution of accuracy, we demonstrated the mean and variance of accuracy for each sleep stage. As shown in Table 5, the average accuracy of the 1-NN classifier in all five sleep stages was close to 50% and the variance was very low. As a result, it can be concluded that the generated data are effective and the SleepGAN we designed performs well in generating EEG signals.

## 4. Conclusions

This study proposed SleepGAN, a novel method for generating EEG epochs and stage transition sequences from a small amount of training data. It has two networks, progressive WGAN-div and RM generator, for data and sequence augmentation. The model integrates stage transition sequences and EEG epochs so that generated data can be utilized in the training set of both CNNs and RNNs. A classical sleep stage classification model DeepSleepNet was trained using the generated data and sequence. When the 30 s epochs of the EEG generated by our SleepGAN were added, the overall accuracy of the model increased by 1%. After both augmented sequence and corresponding augmented data were added, the model reached classification performance with accuracy, Macro F1, and kappa of 83.06%, 74.24%, and 74.78%, respectively. The accuracy of the model increased by 4% from 79.40%, which was obtained by training with only real data. The results show that the EEG epochs and sequences produced by SleepGAN can imitate real data and sequences well and have strong generalization. Overall, SleepGAN is a group of GANs that can efficiently generate high-fidelity EEG epochs using a very small amount of data, which can solve the problem of insufficient training data in automatic sleep stage classification.

## Figures and Tables

**Table 1:** Hyperparameters of generator, discriminator, and training algorithm.

Generator		
Layer	Norm./Act.	Output Size
Latent noise vector	-	1 × 100
Stage 0: 3 × Conv 9	Batch Norm./LreLU (0.05)	32 × 100
Stage 1: Upsampling 3 × Conv 9	- Batch Norm./LreLU (0.05)	32 × 230 32 × 230
Stage 2: Upsampling 3 × Conv 9	- Batch Norm./LreLU (0.05)	32 × 965 32 × 965
Stage 3: Upsampling 3 × Conv 9	- Batch Norm./LreLU (0.05)	32 × 3000 32 × 3000
Conv 9	-/Tanh	1 × 3000
**Discriminator**		
**Layer**	**Act.**	**Output size**
Input signal	-	1 × 3000
3×Conv 9	LreLU (0.05)	32 × 3000
Conv 9	-	1 × 3000
**Training algorithm**		
**General**		
Number of epochs to train per scale		2000
Gamma and milestone of learning rate scheduler		0.1, 1600
Batch size		64
**Generator: Stage n**		
Noise amplitude		0.1
Number of stages N		4
Concurrently trained stages		Last 3 stages
Optimizer: Adam [37]		lr = 0.0005 × 0.1N-n-1 beta1 = 0.5 beta2 = 0.999
Generator inner steps		3
**Discriminator**		
Optimizer: Adam		lr = 0.0005 beta1 = 0.5 beta2 = 0.999
Generator inner steps		3
**Loss**		
WGAN-div loss		K = 2, *p* = 6

**Table 2:** Hyperparameters of stage transition sequence generation.

Model	
Number of memory slots	1
Number of heads	2
Head size	64
Memory size (number of heads × head size)	2 × 64 = 128
Number of layers of MLP in post attention	2
Hidden size of MLP	128
Activation of MLP	ReLU
Training algorithm	
Number of epochs	200
Sequence length	180
Batch size	64
Loss	MLE loss
Optimizer: Adam	lr = 1 × 10^−3^ beta1 = 0.9 beta2 = 0.999
Generator inner steps	3

**Table 3:** Classification performance of training with real data and augmented data.

Test Name	Overall Metrics			Per-Class F1-Score(F1)				
	ACC	MF1	k	W	N1	N2	N3	REM
1-1	0.775	0.663	0.670	0.753	0.285	0.831	0.735	0.694
1-2	0.788	0.693	0.694	0.789	0.319	0.841	0.777	0.731
2-1	0.794	0.701	0.700	0.806	0.375	0.847	0.762	0.716
2-2	0.804	0.717	0.716	0.822	0.371	0.854	0.783	0.753

**Table 4:** Classification results of training with augmented data and augmented sequence.

Test Name	Overall Metrics			Per-Class F1-Score(F1)				
	ACC	MF1	k	W	N1	N2	N3	REM
2-1-1	0.811	0.708	0.719	0.721	0.418	0.850	0.756	0.792
2-1-2	0.814	0.714	0.723	0.741	0.423	0.850	0.741	0.797
2-2-1	0.829	0.735	0.745	0.745	0.438	0.864	0.769	0.844
2-2-2	0.831	0.742	0.747	0.767	0.450	0.864	0.771	0.844

**Table 5:** The average accuracy and variance of accuracy of using 1-NN classifier to evaluate the distribution of data generated by GANs for each sleep stage.

Results	Sleep Stages				
	W	N1	N2	N3	R
Average	52.37%	54.84%	53.74%	57.75%	50.63%
Variance	0.003286	0.01009	0.008936	0.01277	0.000098

## Data Availability

Link to publicly archived datasets SleepEDF: https://www.physionet.org/physiobank/database/sleep-edfx.
